# Acute and long-term effects of psilocybin on energy balance and feeding behavior in mice

**DOI:** 10.1038/s41398-022-02103-9

**Published:** 2022-08-11

**Authors:** Nicole Fadahunsi, Jens Lund, Alberte Wollesen Breum, Cecilie Vad Mathiesen, Isabella Beck Larsen, Gitte Moos Knudsen, Anders Bue Klein, Christoffer Clemmensen

**Affiliations:** 1 Novo Nordisk Foundation Center for Basic Metabolic Research, Faculty of Health and Medical Sciences, University of Copenhagen, Copenhagen, Denmark; 2 Neurobiology Research Unit, Copenhagen University Hospital, Rigshospitalet Copenhagen, Denmark; 3 Faculty of Health and Medical Sciences, University of Copenhagen, Copenhagen, Denmark

**Keywords:** Physiology, Molecular neuroscience, Diseases

## Abstract

Psilocybin and other serotonergic psychedelics have re-emerged as therapeutics for neuropsychiatric disorders, including addiction. Psilocybin induces long-lasting effects on behavior, likely due to its profound ability to alter consciousness and augment neural connectivity and plasticity. Impaired synaptic plasticity in obesity contributes to ‘addictive-like’ behaviors, including heightened motivation for palatable food, and excessive food seeking and consumption. Here, we evaluate the effects of psilocybin on feeding behavior, energy metabolism, and as a weight-lowering agent in mice. We demonstrate that a single dose of psilocybin substantially alters the prefrontal cortex transcriptome but has no acute or long-lasting effects on food intake or body weight in diet-induced obese mice or in genetic mouse models of obesity. Similarly, sub-chronic microdosing of psilocybin has no metabolic effects in obese mice and psilocybin does not augment glucagon-like peptide-1 (GLP-1) induced weight loss or enhance diet-induced weight loss. A single high dose of psilocybin reduces sucrose preference but fails to counter binge-like eating behavior. Although these preclinical data discourage clinical investigation, there may be nuances in the mode of action of psychedelic drugs that are difficult to capture in rodent models, and thus require human evaluation to uncover.

## Introduction

Obesity and addiction share neurobiological and behavioral similarities [1]. An excessive craving for food is perpetuated by the activation of mesolimbic reward circuits, where drugs of abuse also have their effect [2]. This may contribute to an inability to curb eating or a ‘relapse’ in feeding habits, as it becomes difficult to maintain the lowered food intake required to sustain weight loss [3]. Adaptations to neural circuitry mean that in both conditions, behaviors become increasingly compulsive and difficult to correct [2]. Substance use disorders and obesity can to some extent be considered diseases of plasticity; both chronic drug exposure [4] and long-term exposure to a high-fat diet [5] lead to persistent synaptic reorganization. Moreover, many of the genes associated with body mass index (BMI) encode proteins involved in glutamatergic signaling and synaptic plasticity [6]. These parallels suggest that similar therapeutic approaches could be applied to the treatment of obesity and addictive disorders, and psilocybin presents a promising candidate due to its remarkable ability to alter synaptic architecture. Psilocybin, and other serotonergic psychedelics, have re-emerged as therapeutics for neuropsychiatric disorders, where the development of new pharmacological treatments has stalled due to a lack of long-term efficacy for treatment-resistant patients [7]. Psilocybin is systemically metabolized to psilocin which activates the serotonin 2A receptor (5-HT_2A_R), altering perception to induce its hallucinogenic effect [8]. The downstream consequence is the promotion of functional and structural neuroplasticity [9], facilitating enduring effects on behavior, for example, long-lasting improvement in depressive symptoms [10].

As a serotonin receptor agonist, psilocybin is especially promising for the treatment of obesity. The natural ligand for 5-HT_2A_R, serotonin, acts on several receptors to have a wide range of biological effects, including modulating food intake [11, 12]. Clinical studies have found that cerebral 5-HT_2A_R binding is positively correlated to body mass index (BMI) [13] and high pre-surgical 5-HT_2A_R binding (as well as the change in 5-HT_2A_R binding) correlates with weight loss after gastric bypass surgery [14]. Serotonin integrates metabolic signals that convert energy status but also has an effect on reward-related feeding [15]. Decreased serotonin signaling is associated with obesity, and modulation of the serotonergic system affects food intake [16–18]. Early work identified that alongside d-amphetamines, classic serotonergic psychedelic lysergic acid diethylamide (LSD) and psilocybin reduce food consumption in rats and dogs [19, 20]. Additionally, activation of 5-HT_2A_R has anti-inflammatory effects in a mouse model of cardiovascular disease [21]. Self-reported data from psychedelic users shows that one-time use of a classic psychedelic reduces the risks of becoming obese [22]. Taken together, these results imply a potential for psilocybin to be therapeutically promising for the treatment of obesity.

In the present study, we used mouse models of genetic obesity, diet-induced obesity, and binge-eating disorder to examine the effect of psilocybin on body weight and food intake. A single high dose of psilocybin did not have an effect on body weight or food intake in diet-induced obese (DIO) mice or genetic mouse models of obesity. Microdosing also failed to correct obesity in DIO mice and in obese melanocortin 4 receptor (MC4R)-deficient mice. No effect was observed on binge-eating behavior. Explorative RNA sequencing revealed only subtle transcriptional changes in the hypothalamus, 3 h or 4 weeks after a single injection with psilocybin. In contrast, 3 h following psilocybin treatment more than 1500 transcripts were significantly changed in the prefrontal cortex (PFC). A single administration of psilocybin, however, did not produce any long-lasting transcriptional changes in the PFC. In summary, the presented preclinical evidence is not in favor of the use of psilocybin for the management of obesity. However, further clinical investigation might shed light on the use of psychedelics for the treatment of human obesity or obesity-related disorders.

## Materials and methods

### Animals

Wild type male C57BL/6J mice (Janvier, FR), male leptin-deficient *ob/ob* mice (The Jackson, Laboratory, stock no. 00632), and male melanocortin-4 receptor knockout mice (The Jackson Laboratory, stock no. 032518) were used. Except otherwise stated, mice were group-housed. All mice were kept under normal room temperature (22 °C) on a standard 12 h light–dark cycle, with ad libitum access to water and a chow diet (Altromin D30, Brogaarden). Diet-induced obese mice were switched from a chow diet to a high-fat diet at 8 weeks of age (D12331; Research Diets, New Brunswick, NJ, USA) in order to facilitate the development of adiposity. Prior to experiments, mice were acclimatized to housing conditions for a minimum of one week, and sham injected with isotonic saline in the 3 days preceding study start. Mice used in the head-twitch study were not sham-injected. Sample sizes were selected on the basis of previous experiments using similar methods. In experiments with lean C57BL/6J mice, mice were block-randomized into groups. Where diet-induced obese C57BL/6J mice, *ob/ob* mice, or MC4R mice were used, mice were stratified into groups to ensure body weight was matched. All in vivo experiments were carried out in accordance with regulations regarding the care and use of experimental animals, and the Danish Animal Experimentation Inspectorate approved the experimental procedures (2018-15-0201-01457).

### Drugs

Psilocybin was purchased from Mikrolab Aarhus (Højberg, DK) or obtained from Rigshopitalet, Denmark (the latter source only used for pilot studies referenced in supplementary materials). Liraglutide (Victoza^®^, Novo Nordisk, Bagsvaerd, DK). Fluoxetine hydrochloride (Sigma-Aldrich, #F132-50MG). All drugs were diluted in isotonic saline.

### Head-twitch response

Head-twitch response was evaluated in 17-week-old male C57BL/6J mice on a chow diet (group-housed). Mice were randomized into treatment groups (*n* = 4–5) and received a single intraperitoneal injection of 0.3 mg/kg psilocybin, 1 mg/kg psilocybin, 3 mg/kg psilocybin, or isotonic saline. Following injection, each mouse was placed into its own new cage with bedding. Two Logitech C920 HD webcams were mounted onto cages, and the behavior of each mouse was recorded for 20 min. The number of head twitches (defined as ‘rapid paroxysmal rotational movement of the head’) [23] was counted by two observers blinded to the experimental conditions.

### Open field test

Locomotor activity was assessed in an open field arena (w: 50 cm, h: 50 cm) under normal lighting conditions. Fourteen-week-old male C57BL/6J mice (maintained on a chow diet, single housed) received a single intraperitoneal injection of either 3 mg/kg psilocybin or isotonic saline (*n* = 8 per group). Thirty minutes post-injection, mice were placed in the open field arena, and their locomotor activity was recorded for 20 min with a ceiling-mounted Logitech C920 camera. Distance moved and time spent in the center zone was calculated using a computer-based tracking system (Ethovision XT, Netherlands).

### Voluntary running

Ten-week-old male C57BL/6J mice on a chow diet were single-housed in cages equipped with running wheels (23 cm in diameter, Techniplast I). Running distance was measured by a computer (Sigma Pure 1 Topline 2016, D). After 5 days of familiarization with the wheels, the running distance was measured on days −2 and −1. These data were used to create two groups of mice with similar baseline running distances (*n* = 8 per group). On day 0, two hours prior to the dark phase, mice were injected intraperitoneally with either psilocybin or isotonic saline, and subsequent running distance was measured 4, 6, 8, 16, and 24 h after injection and again on days 2, 3, 4, and 5. Two samples were removed from the psilocybin group (day 2 and day 5) as the system failed to detect any activity.

### Metabolic assessment

To assess the effect of psilocybin on whole-body energy expenditure and substrate utilization, 9-week-old male C57BL/6J mice were single-housed in indirect calorimetry chambers (TSE System, Germany). Mice were habituated to the indirect calorimetry chambers from day −7 to day 0. During these days, body weight was measured on a daily basis in the afternoon, and data on food intake, water intake, locomotor activity, oxygen consumption, and respiratory exchange ratio were automatically collected every 20 min. These data were used to create two groups of mice (*n* = 8 per group) with similar average body weights and whole-body metabolic profiles. Mice were sham injected at days −3, −2, and −1. The collection of experimental data started on day −1. On day 0, 45 min before the onset of the dark phase, mice in one group were injected intraperitoneally with psilocybin (3 mg/kg) while mice in the other group were injected with isotonic saline. The automatic 20 min-interval measurements of oxygen consumption, carbon dioxide release, and food intake continued for 4 days. On day 5, the chow diet was switched to a high-fat diet, and the automatic measurements were continued until the experiment was terminated on day 8. Two animals were excluded from the analysis of water intake due to leaky water bottles.

### Body temperature

Body temperature was assessed via inserting a probe (BIO-TK8861, Bioselab, France) into the rectum of 8-week-old male C57BL/6J mice (double-housed) on a chow diet. On the day of the experiment, four hours after the onset of the light phase, mice were treated with a single intraperitoneal injection of 3 mg/kg psilocybin or isotonic saline (*n* = 8 per group). Rectal temperature was measured before injection (0 min) and at 15, 30, 60, 120, 180, 360 min, and 24 h following treatments.

### Blood biochemistry

To assess the circulating level of corticosterone, cholesterol, triglycerides, and insulin, 8-week-old male C57BL/6J mice (double-housed) on a chow diet were randomized to treatment with a single intraperitoneal injection of either 3 mg/kg psilocybin or isotonic saline (*n* = 8 per group). Blood samples were drawn (from the tail tip) before treatment (0 h) and again, 3 and 24 h following treatment. Samples were immediately centrifuged at 3000 rpm at 4 °C for 10 min (to obtain plasma) and stored at −20 °C for analysis. Blood glucose was measured in a drop of whole blood at each time point using a handheld glucometer (Contour XT, Bayer). Plasma corticosterone was measured at (0, 3, and 24 h) using the Detect X Corticosterone assay kit (Arbor Assays, USA). Plasma cholesterol, triglycerides, and insulin were measured only in 24 h samples using Infinity Cholesterol and Infinity Triglycerides kits (Thermofisher Scientific) and Ultrasensitive Mouse Insulin Elisa Kit (Mercodia).

### Food intake and body weight studies

Male DIO C57BL/6J mice at 46 weeks of age (maintained on a high-fat diet) were single-housed and divided (based on body weight) into two groups (*n* = 8 per group). Mice received a single intraperitoneal injection of either 3 mg/kg psilocybin or isotonic saline. In the 12 subsequent days, food intake and body weight were measured daily, before the onset of the dark phase.

A similar study was conducted in 21-week-old leptin-deficient *ob/ob* mice (single and double housed) that received either a single intraperitoneal dose of 3 mg/kg psilocybin (*n* = 9) or isotonic saline (*n* = 7). Food intake and body weight were measured daily, before the onset of the dark phase, for 7 days following injection.

### Dietary intervention study

To assess whether psilocybin enhances diet-induced weight loss, a new experimental paradigm was designed. Twenty-four single-housed male DIO C57BL/6J mice at 47 weeks of age were divided into three groups based on body weight (i = 8 per group) as follows:

*Group 1***:** Ad libitum access to a chow diet and high-fat diet for the duration of the experiment, and were treated at day 0 with a single intraperitoneal injection of isotonic saline. This group served as a control for baseline food intake and body weight following injection stress. One mouse in group 1 was excluded from the analysis due to excessive weight loss. *Group 2***:** High-fat diet was removed on day 0 of the experiment, in order to stimulate ‘diet-induced weight loss’, and mice were injected with 3 mg/kg psilocybin, to identify whether psilocybin treatment could potentiate the diet-induced weight loss. The high-fat diet was returned seven days after injection. *Group 3***:** High-fat diet was removed on day 0 of the experiment, in order to stimulate ‘diet-induced weight loss’ as in group 2, but mice were injected with isotonic saline. The high-fat diet was returned 7 days after injection. This group served as a control to determine psilocybin’s effect on body weight following the dietary intervention, and body-weight regain. Two data points were removed from the analysis due to scale mis-readings at the time of measurement.

### Binge eating paradigm

A binge eating paradigm was established according to an existing protocol [24]. Twenty-one-week-old male C57BL/6J mice (single-housed, on a chow diet) were divided into one of two experimental groups termed ‘continuous’ (*n* = 20) or ‘intermittent’ (*n* = 19) as follows:

*Group 1, ‘Intermittent’***:** Ad libitum access to chow diet and high-fat diet for 48 h, after which the high-fat diet was removed for 5 days (mice had ad libitum access to chow diet). The high-fat diet was returned to the ‘intermittent’ group for 24 h, during which chow and high-fat diet intake were measured at 2.5 and 24 h. The high-fat diet was removed for 6 days (during which, mice had ad libitum access to chow diet)—constituting the first binge cycle. A second binge cycle was initiated immediately after the first. Cycles of short-term exposure to a high-fat diet establish a ‘binge-like’ phenotype in the ‘intermittent’ group. *Group 2, ‘Continuous’***:** Ad libitum access to a chow diet and high-fat diet for the duration of the study (3 weeks).

Following two binge cycles, mice were randomized to groups for pharmacological evaluation of the model. On the day of the experiment, five mice from the ‘continuous’ group were selected as controls, to establish baseline intake of chow and high-fat diet with ad libitum access. These mice received an intraperitoneal injection of isotonic saline. Eighteen mice from the ‘intermittent’ group had access to a chow diet only in the 6 days preceding the experiment. On the day of the experiment, the selected mice received either a single intraperitoneal injection of fluoxetine (30 mg/kg), psilocybin (3 mg/kg), or isotonic saline (*n* = 6 per group). Thirty minutes following treatment, mice in the intermittent group were given 24 h access to a high-fat diet and chow. Food intake was measured in all groups at 2.5 and 24 h after injection.

### Sucrose preference test

Fourteen-week-old chow-fed male C57BL/6J mice (single-housed) were acclimated to the presence of two water bottles in their home cage, for 24 h. The water bottles were weighed to obtain a baseline intake. On the test day, 2 h prior to the onset of the dark phase, bottles containing fresh water and a 2% sucrose solution were added to the home cages, and mice were subsequently injected intraperitoneally with psilocybin (3 mg/kg, *n* = 10) or isotonic saline (*n* = 10). Mice were free to consume liquid from either bottle for 24 h, after which the bottles were weighed to measure consumption. The study was repeated the next day, with the bottles reversed in position (in the cage) to account for side preference. Sucrose and water intake over two study days were averaged.

The sucrose preference test was repeated in the second cohort of chow-fed male mice. Mice received daily intraperitoneal injections of psilocybin (0.3 mg/kg) or isotonic saline (*n* = 8 per group) for 5 days, and food intake and bottle weight were recorded daily.

### Bulk RNA sequencing

Ten-week-old chow-fed male C57BL/6J mice (group-housed) were randomized into one of four treatment groups (*n* = 8 per group) as follows:

*Group 1*: A single intraperitoneal injection of 3 mg/kg psilocybin, followed by tissue harvest 3 h after treatment. *Group 2*: A single intraperitoneal injection of isotonic saline followed by tissue harvest 3 h after treatment. *Group 3***:** A single intraperitoneal injection of 3 mg/kg psilocybin followed by tissue harvest 4 weeks after treatment. *Group 4***:** A single intraperitoneal injection of isotonic saline followed by tissue harvest 4 weeks after treatment.

On the day of the experiment, mice received a single intraperitoneal injection according to their grouping and were sacrificed (decapitation) according to the above schedule. The prefrontal cortex and whole hypothalamus were immediately dissected out and flash frozen on dry ice. Mice were euthanized in the morning, 4 h after the onset of the light phase, to ensure minimal diurnal variation in gene expression. Dissected brain regions were stored at −80 °C until isolation of RNA was performed.

### Gene expression analysis (qPCR)

Brown adipose tissue (BAT) was dissected from all mice used in the bulk RNA seq study and stored at −80 °C until analyzed. For gene expression analysis, total BAT RNA was isolated according to manufacturer's protocol, using RNeasy Minikit (Qiagen, #74106). After quantification of RNA with NanoDrop 2000 (Thermo Fisher), cDNA was synthesised by mixing RNA with FS buffer, DTT (Thermo Fisher), and Random Primers (Sigma-Aldrich) and incubated for 3 min at 70 °C. Superscript III (Thermo Fisher) and RNase out and dNTPs were added, before samples were placed into a thermocycler for 5 min at 25 °C, 60 min at 50 °C and 15 min at 70 °C. cDNA was diluted 1:100 and stored at −20 °C until further processing. qPCR was performed using SYBR green (Thermo Fisher), and gene expression analysis normalized to housekeeping gene 36b4, according to the delta-delta Ct method.

### Transcriptomic analysis by RNA sequencing

Total RNA was isolated using an RNeasy mini kit (Qiagen, #74106) according to the manufacturer’s protocol. Messenger RNA sequencing libraries were prepared using the Illumina TruSeq Stranded mRNA protocol (Illumina). Poly-A containing mRNAs were purified by poly-T attached magnetic beads, fragmented, and cDNA was synthesized using SuperScript III Reverse Transcriptase (Thermo Fisher Scientific). cDNA was adenylated to prime for adapter ligation and after a clean-up using AMPure beads (Beckman coulter), DNA fragments were amplified using PCR followed by a final clean-up. Libraries were quality-controlled using a Fragment Analyzer instrument (Agilent Technologies) and subjected to 52-bp paired-end sequencing on a NovaSeq 6000 (Illumina). A total of 2.7 billion reads were generated.

### Bioinformatic analyses

Fastq files were generated using bcl2fastq2 v. 2.20.0, then reads were aligned to the GRCm38 primary assembly using STAR v. 2.7.2b [25] against a reference built using the GENCODE vM25 gene model [26]. Aligned reads were counted against the same gene model using featureCounts v. 1.6.2 [27] counting only reads where both ends mapped to the same strand. The separation between tissues and homogeneity within tissues was visually inspected using MDS plots. Two samples were excluded for having almost no reads and one sample was excluded for being heavily enriched in genes related to wound healing and immune system processes. Visual inspection revealed an extraction pool to separate samples. Genes with sufficient counts were selected using the filterByExpr function from the edgeR v. 3.30.2 [28] package and were transformed using the voomWithQualityWeights function [29]. Differential expression was calculated using limma v. 3.44.0 using a model of the form *~group* + *extraction_pool*, where *group* encoded tissue, treatment, and timepoint and *extraction_pool* encoded the extraction pool. Animal-specific effects were modeled using the duplicateCorrelation function which is part of limma [30]. Contrasts were specified as described in the limma manual. Gene ontology and reactome enrichments were found using the camera function [31]. All plots were generated using ggplot2 [32].

### Statistical analyses

Statistical analyses were performed in GraphPad Prism version 9. All experiments were performed once. Statistics for each experiment can be found in respective figure legends. All data are presented as mean ± SEM and all *P* values ≤ 0.05 were considered statistically significant. *P ≤ 0.05, **P ≤ 0.01, ***P ≤ 0.001, ****P ≤ 0.0001.

## Results

### Establishing a psychoactive dose of psilocybin for metabolic studies

**Fig. 1:**
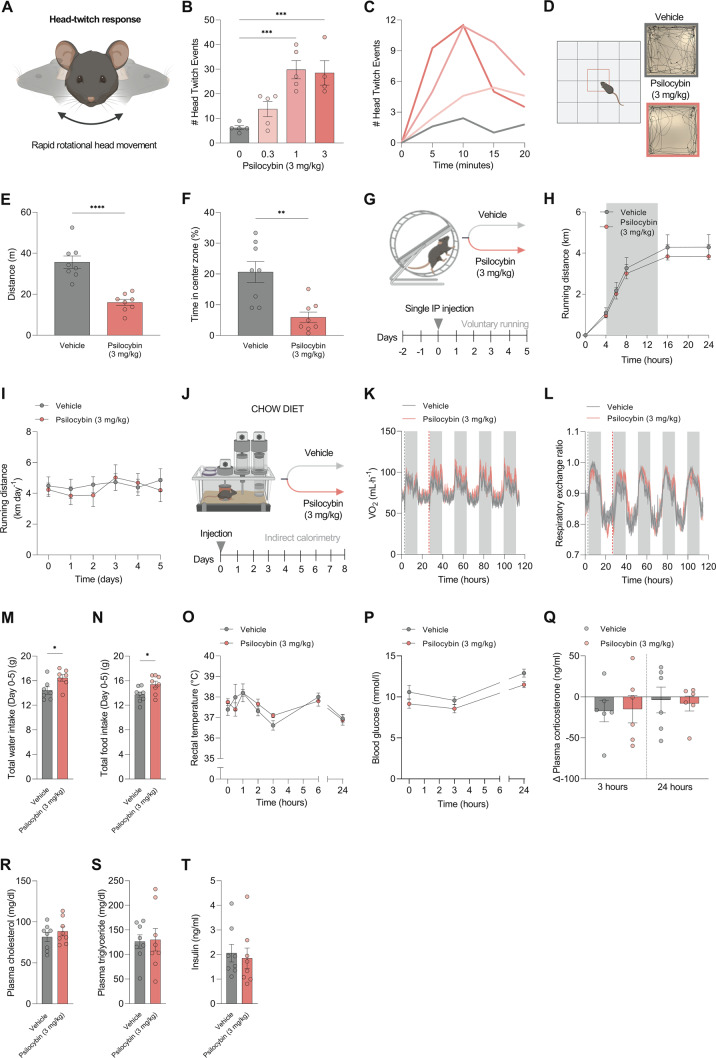
A single high dose of psilocybin does not have an effect on basal physiology in chow-fed mice. **A** Schematic overview of the head-twitch response study (characterized by a rapid side-to-side head movement), was used to inform the dose for further experiments. **B** Total number of head-twitches at each dose. **C** Time course of head-twitch events. **D** Schematic of open field test (OFT), and representative traces for a vehicle-treated and psilocybin-treated (3 mg/kg) mouse. **E** Distance traveled in the assessment of exploratory behavior in the OFT, 30 min after administration with psilocybin (3 mg/kg) or vehicle (*n* = 8 per group). **F** Percentage of time spent in the center zone. **G** Schematic overview of voluntary wheel running experiment. **H** Effect of a single high dose of psilocybin on voluntary wheel behavior (24 h), (*n* = 7–8 per group). **I** Running distance in the five days following injection with psilocybin (3 mg/kg; *n* = 8) or vehicle (*n* = 7). **J** Schematic of indirect calorimetry study of mice treated with psilocybin (3 mg/kg) or vehicle (*n* = 8 per group). **K** VO_2_. Red dashed line indicates the day of injection. **L** Respiratory exchange ratio. Red dashed line indicates day of injection. **M** Water intake (cumulative) after five days in metabolic cages. **N** Cumulative food intake after 5 days in metabolic cages. **O** Rectal temperature 0, 15, 30 min, 1, 3, 6, and 24 h after administration of psilocybin (3 mg/kg) or saline (*n* = 8 per group). **P** Blood glucose at 0 min, 3, and 24 h following administration of psilocybin (3 mg/kg) or vehicle (*n* = 8 per group). **Q** Change in plasma corticosterone 3 and 24 h following administration of psilocybin (3 mg/kg) or vehicle (*n* = 6 per group). **R** Total plasma cholesterol 24 h after administration of psilocybin (3 mg/kg) or vehicle (*n* = 8 per group). **S** Plasma triglycerides 24 h after administration of psilocybin (3 mg/kg) or vehicle (*n* = 8 per group). **T** Plasma insulin 24 h after administration of 3 mg/kg or vehicle (*n* = 8 per group). Data analyzed by one-way ANOVA with Dunnett’s test (**B**), unpaired two-tailed *t*-test (**E**, **F**, **M**, **N**, **Q**, **R**, **S**, **T**), repeated measures two-way ANOVA with Bonferroni’s multiple comparison test (**H**, **O**, **P**). Mixed effects analysis with Bonferroni’s multiple comparison test (**I**; missing values as one mouse failed to run on two occasions). Data are presented as mean ± SEM. *P < 0.05, **P < 0.01, ***P < 0.001, ****P *<* 0.0001.

To test the bioactivity of psilocybin and determine an appropriate dose for assessing the metabolic effects of psilocybin in mice, we measured the head-twitch response following a single administration of 0.3 mg/kg psilocybin, 1 mg/kg psilocybin, 3 mg/kg psilocybin or vehicle (isotonic saline). The head twitch response is a rapid rotational head movement [23], stereotypical in rodents following activation of the serotonin 5-HT_2A_R with psychedelics (Fig. 1A). Consistent with existing literature, 1 and 3 mg/kg induced a greater number of head twitches than other doses (Fig. 1B, C) [33]. To further confirm that the 3 mg/kg dose was sufficient for inducing a ‘psychoactive’ effect in mice, we performed an open-field test. Mice were injected intraperitoneally with 3 mg/kg psilocybin or saline, and their behavior was recorded for 20 min after treatment (Fig. 1D). Psilocybin-treated mice exhibited significantly less movement than mice treated with saline (Fig. 1E), and spent significantly less time in the center zone (Fig. 1F). The increase in thigmotaxis (time spent close to the walls) following psilocybin-treatment could be interpreted as anxiogenic [34], but in this instance, seems to relate to reduced total locomotion. Based upon these data and existing literature (which supports the use of 1–3 mg/kg in rodents [35], we selected 3 mg/kg as the dose for subsequent metabolic in vivo studies. Selection of dose was supported by dose-titration pilot studies in DIO C57BL6/J mice, showing that a single subcutaneous injection of psilocybin at 0.3, 1, and 3 mg/kg had a marginal effect on sub-chronic weight loss and no difference on food intake (Fig. S1A, B).

### Psilocybin does not affect energy metabolism in mice

In a study assessing alcohol consumption following psychedelic use, alongside recidivism, participants reported an increase in exercise [36]. To examine the effect of psilocybin on voluntary physical activity, male C57BL/6J mice (maintained on a chow diet) were adapted to running wheel access, and injected with a single dose of psilocybin (3 mg/kg) or vehicle (Fig. 1G). Consistent with existing literature [37], mice did not exhibit differences in acute or chronic treadmill running following administration of psilocybin (Fig. 1H, I). Human data suggest transient effects of psilocybin on blood pressure and on the hypothalamic-pituitary-thyroid (HPT) axis [38], indicating psilocybin might also regulate metabolic rate. Whether these changes coincide with increased energy expenditure is unknown. To study psilocybin’s effects on whole-body energy metabolism, we employed indirect calorimetry metabolic cages (Fig. 1J). In the 5 days following a single injection with psilocybin, energy expenditure and substrate metabolism were similar between groups (Fig. 1K, L). The mice injected with psilocybin displayed a slightly increased food intake and water intake whilst maintained on a chow diet (Fig. 1M, N). The observed increase in water intake was also observed following a 1 mg/kg dose of psilocybin to DIO mice in metabolic cages (Fig. S1E). Serotonin 5-HT_2A_ receptors are implicated in thermoregulation and the 5-HT_2A_R agonist lysergic acid diethylamide (LSD) causes hyperthermia in humans [39]. Analysis of brown adipose tissue in mice injected with a single dose of psilocybin (3 mg/kg) showed no differences in ‘thermogenic’ gene expression compared to control-treated counterparts (Supplementary Fig 1F, G). The physiological effects of psilocybin in humans are limited, but in one study, psilocybin administration did not have an effect on body temperature [38]. Similarly, we observed no effect on body temperature at the selected dose (Fig. 1O). Blood glucose and plasma corticosterone were recorded at baseline, 3 and 24 h (in two separate cohorts of mice). To date, no effect of psilocybin on blood glucose has been observed in humans, but the 5-HT_2A_R agonist—DOI (2,5-dimethoxy-4-iodophenyl)−2-aminopropane) restores glucose homeostasis in apolipoprotein E (ApoE) knockout mice [40, 41]. Here, we demonstrate no significant effect of psilocybin on blood glucose or plasma corticosterone (Fig. 1P, Q). In contrast, plasma cortisol is increased following the administration of both psilocybin and LSD in humans [38, 42]. We report no effect of psilocybin on plasma levels of cholesterol, triglyceride, or insulin (Fig. 1R–T).

### Psilocybin does not affect body weight or food intake in mouse models of obesity

**Fig. 2:**
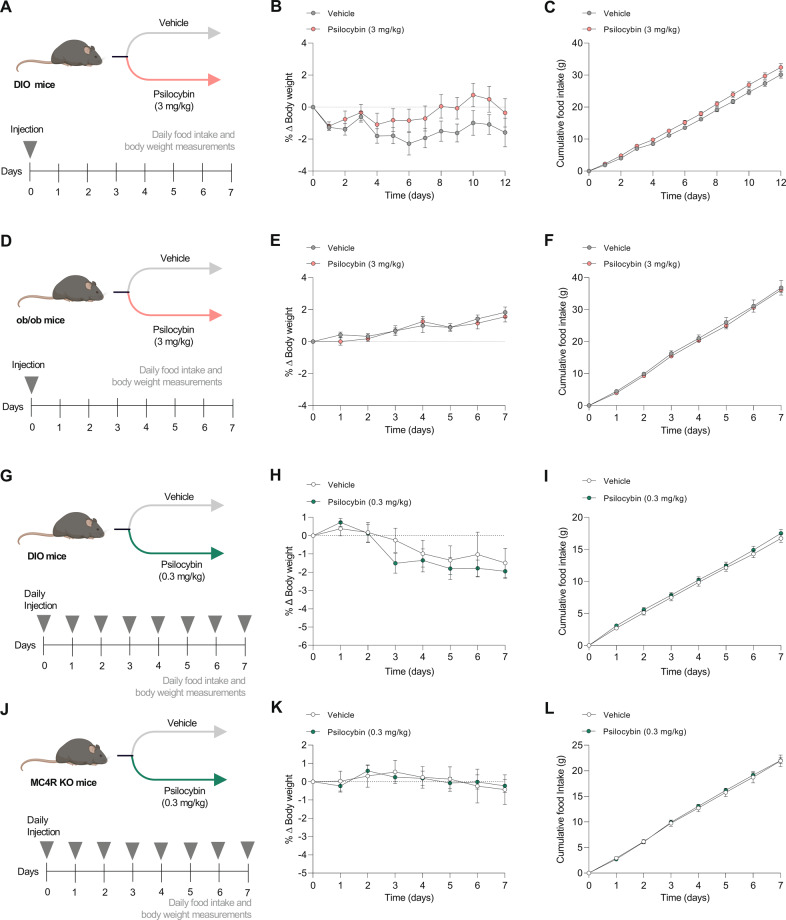
A single high dose or microdosing psilocybin does not have an effect on body weight or food intake in mouse models of obesity. **A** Schematic for single injection study in diet-induced obese (DIO) mice (*n* = 8 per group). **B** Body weight in the 12-day period following injection of psilocybin (3 mg/kg) or vehicle. **C** Cumulative food intake in the 12 days following injection of psilocybin or vehicle. **D** Schematic for single injection study in ob/ob mice (*n* = 7–9 per group). **E** Body weight. **F** Cumulative food intake. **G** Schematic for microdosing study in DIO mice. Mice were injected daily with psilocybin (0.3 mg/kg; *n* = 7) or vehicle (*n* = 6). **H** Body weight following daily injection of psilocybin (0.3 mg/kg) or vehicle for 7 days (*n* = 6/7 per group). **I** Food intake. **J** Schematic for microdosing study in MC4R KO mice (*n* = 8 per group). **K** Body weight. **L** Food intake. Data are presented as mean ± SEM (analyzed by RM two-way ANOVA with Bonferroni’s test).

In humans, drugs that increase central serotonin have a marked anorectic effect [43], whilst BMI is correlated with cerebral 5-HT_2A_R expression [13] Mice treated with the selective 5-HT_2A_R agonists DOI and TCB-2 ((4-Bromo-3,6-dimethoxybenzocyclobuten-1-yl) methylamine hydrobromide) exhibit hypophagia [44, 45]. Although the 5-HT_2C_ receptor is more widely studied in the context of energy balance, there are indications that the 5-HT_2A_R plays an underappreciated role—especially as it has been shown to be important for mediating the weight-lowering effect of GLP-1 [46]. In the present study, we wanted to examine whether psilocybin, as an agonist of 5-HT_2A_Rs, may have observable effects on body weight or food intake in a mouse model of diet-induced obesity (Fig. 2A). Following peripheral injection of 3 mg/kg psilocybin, no difference was observed in body weight or food intake in the 12 days following injection (Fig. 2B, C). On the final day of the experiment, an intraperitoneal glucose tolerance test was performed to investigate whether psilocybin has long-term effects on glucose homeostasis, but there was no difference compared to vehicle-treated mice (Fig. S2A, B).

Similarly, we wanted to assess the effect of psilocybin in leptin-deficient *ob/ob* mice, a commonly used genetic model of obesity (Fig. 2D) [47]. *Ob/ob* mice are reported to have reduced 5-HT transporter mRNA in the dorsal raphe nucleus—which is associated with a ‘depressive’ phenotype, alongside impaired metabolism [48]. Also, administering dexfenfluramine (which increases levels of extracellular serotonin) to *ob/ob* mice attenuates weight gain on a moderate-fat diet [49]. In contrast, we demonstrate that a single dose of 3 mg/kg psilocybin does not influence the body weight or food intake of *ob/ob* mice on a high-fat diet (Fig. 2E, F).

In addition to the single-injection protocol, we investigated the effect of a microdosing-like strategy on body weight (Fig. 2G). Psychedelic microdosing has been popularized for its benefits on creativity, problem-solving ability, and energy levels [50, 51]. In a separate cohort of DIO mice, intraperitoneal injection of psilocybin (0.3 mg/kg) or vehicle was administered daily. No significant effect was observed on either body weight or food intake using this approach (Fig. 2H, I). Previous work identified that serotonin-mediated hypophagia requires downstream activation of melanocortin 4 receptors (MC4R) [12]. A microdosing-like approach was also applied to an MC4R KO mouse model (Fig. 2J). In agreement with studies in DIO mice and *ob/ob* mice, systemic administration of psilocybin did not alter food intake or body weight in MC4R KO mice (Fig. 2K, L).

### Psilocybin has no effect on body weight or food intake in combination with dietary or pharmacological interventions

**Fig. 3:**
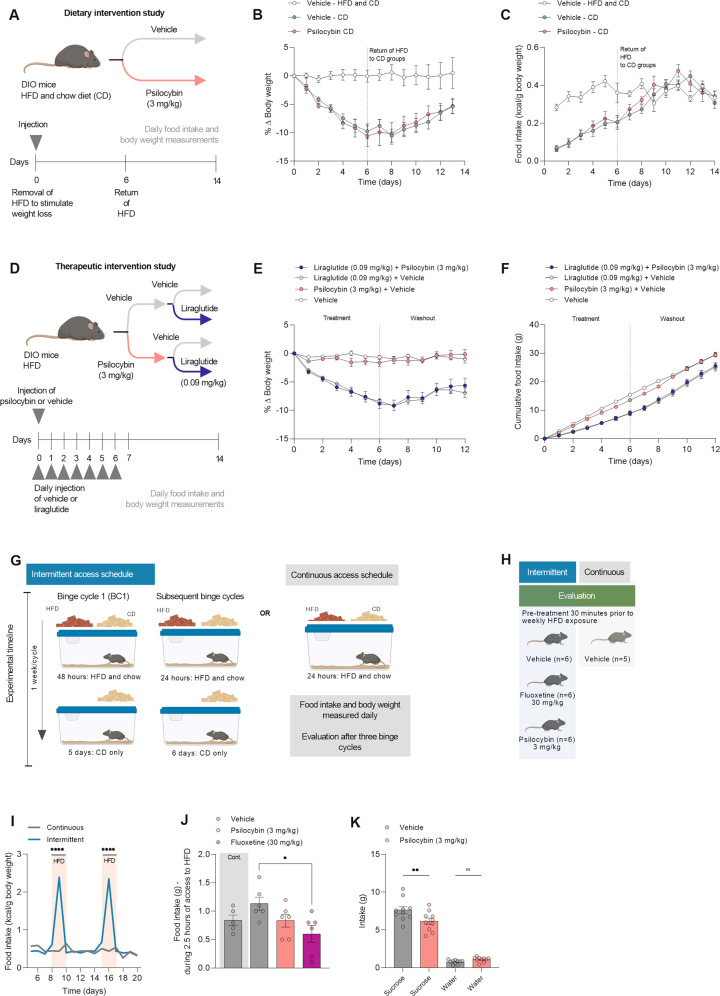
Psilocybin displays no benefits in combinatorial therapeutic paradigms, and has no effect on binge-eating like behavior, but a single high dose of psilocybin reduces sucrose preference in mice. **A** Schematic overview of dietary intervention study (*n* = 8 per group). **B** Body weight of mice in the 14-day period following injection. **C** Caloric intake following a single injection of psilocybin (3 mg/kg) or vehicle alongside dietary intervention. **D** Schematic for therapeutic intervention study (*n* = 8 per group). Diet-induced obese mice were injected with psilocybin (3 mg/kg) and liraglutide (0.09 mg/kg), liraglutide (0.09 mg/kg), and vehicle, or vehicle only. **E** Body weight. **F** Cumulative food intake. **G** Schematic showing set up of binge eating like behavior model. First binge cycle—mice in the intermittent group had 48-h free choice access to standard chow and high-fat diet. Successive binge cycles (binge cycle 2 and 3): mice in the intermittent group had free-choice access to standard chow for 24 h, following 6 days with ad libitum access to a chow diet. **H** Schematic showing pharmacological intervention in the binge-eating model. Mice were cycled through 3 binge cycles, and pharmacological intervention occurred in the 3rd cycle. Following pre-treatment with psilocybin (3 mg/kg), fluoxetine (30 mg/kg), or vehicle, mice in the intermittent access group received a high-fat diet for 24 h. **I** Establishment of binge eating-like behavior in a cohort of mice. **J** Food intake 2.5 h following access to HFD was recorded in each group. **K** Sucrose preference test after treatment with psilocybin (3 mg/kg) or vehicle (*n* = 10) per group. **L** Sucrose consumption after daily administration of psilocybin or vehicle. Data was analyzed by mixed-effects analysis (missing values due to erroneous food scale measures) with Bonferroni’s multiple comparison test (**I**, **J**) and one-way ANOVA and Bonferroni’s multiple comparison test (**E**, **K**) and unpaired *t*-test (**L**). Data are presented as mean ± SEM. *P < 0.05, **P < 0.01, ***P < 0.001, ****P *<* 0.0001.

Studies that assess the potential of psychedelic drugs as therapies for depression and addiction are increasingly using a combination approach, e.g. psilocybin treatment in addition to cognitive behavioral therapy. Psilocybin is thought to enhance ‘cognitive flexibility’—facilitating revision of long-held patterns of behavior [52]. In such a paradigm, psilocybin acts to ‘prime’ the brain, increasing the efficacy of the secondary intervention. In the present study, we wanted to investigate whether psilocybin could provide the same neural flexibility in response to a dietary intervention (switch from HFD to chow only), on the same day as receiving a dose of psilocybin (Fig. 3A). Thus, the high-fat diet-fed obese mice were exposed to a low-fat diet-induced weight loss paradigm. After weight loss was achieved, the mice were returned to the high-fat diet in order to examine whether psilocybin has the ability to facilitate enduring changes following the dietary intervention. In this experiment, mice that received psilocybin did not maintain the diet-induced weight loss more successfully than vehicle-treated mice (Fig. 3B, C). We performed a similar combination intervention study, using the weight-lowering agent, liraglutide (GLP-1 receptor agonist), alongside psilocybin (Fig. 3D). Some of the weight-lowering effects of GLP-1 receptor agonists have been attributed to interaction with 5-HT_2A_Rs, but this idea has also been contended [46, 53]. Diet-induced obese mice received a single injection of psilocybin (or vehicle), alongside a daily injection of liraglutide (or vehicle). Mice dosed with liraglutide lost 9% body weight following 7 days of treatment, but this effect was not augmented by co-administration of psilocybin (Fig. 3E, F).

### Psilocybin does not alter binge-eating behavior, but reduces sucrose preference in lean mice

Early research into psychedelics as medicine showed promise for the use of psilocybin as a treatment for tobacco and alcohol dependence [54, 55]. A repeat study in a mouse model of alcohol relapse failed to replicate this benefit, but recent work identified that psilocybin targets a neural circuit involved in craving, highlighting its potential as a treatment of addiction [56, 57]. No studies to date have evaluated the effect of psilocybin on compulsive feeding behavior. To assess whether psilocybin could have an effect on binge-eating behavior, we used a previously established experimental paradigm to stimulate binge-like eating behavior in a cohort of lean mice, without causing excessive stress [58] (Fig. 3G). Once the binge-eating behavior was established (Fig. S3A, B), we tested the pharmacological potential for psilocybin to reverse the phenotype (Fig. 3H). Fluoxetine hydrochloride was used as a positive control, as it effectively ameliorates binge-like eating behavior in mice [59] (Fig. 3I). After successfully establishing the binge-eating phenotype in two consecutive cycles (Fig. 3A–C), mice were dosed with either fluoxetine, psilocybin or saline during a third binge cycle. Administration of fluoxetine hydrochloride did ameliorate the binge-eating phenotype, but a 3 mg/kg dose of psilocybin failed to do so (Fig. 3J). To determine whether psilocybin could alter sweet taste preference, a sucrose preference test was performed. Psilocybin did lower sucrose preference following a single injection; mice treated with psilocybin drank significantly less sucrose without reducing their water intake (Fig. 3K). A reduction in sucrose preference could be indicative of anhedonia [60], or supportive of the notion that psilocybin might lower the hedonic value of rewarding stimuli [57]. If the latter is true, psilocybin may be of benefit for treating compulsive-reward-seeking behavior or excessive consumption of sweet foods. Repeated administration of 0.3 mg/kg psilocybin did not have an effect on sucrose preference in lean mice (Fig. S3C-F), which suggests that a high dose regimen would be necessary to achieve a reduction in food-motivated reward seeking.

### Psilocybin causes acute transcriptional changes in the prefrontal cortex, but not in the hypothalamus

**Fig. 4:**
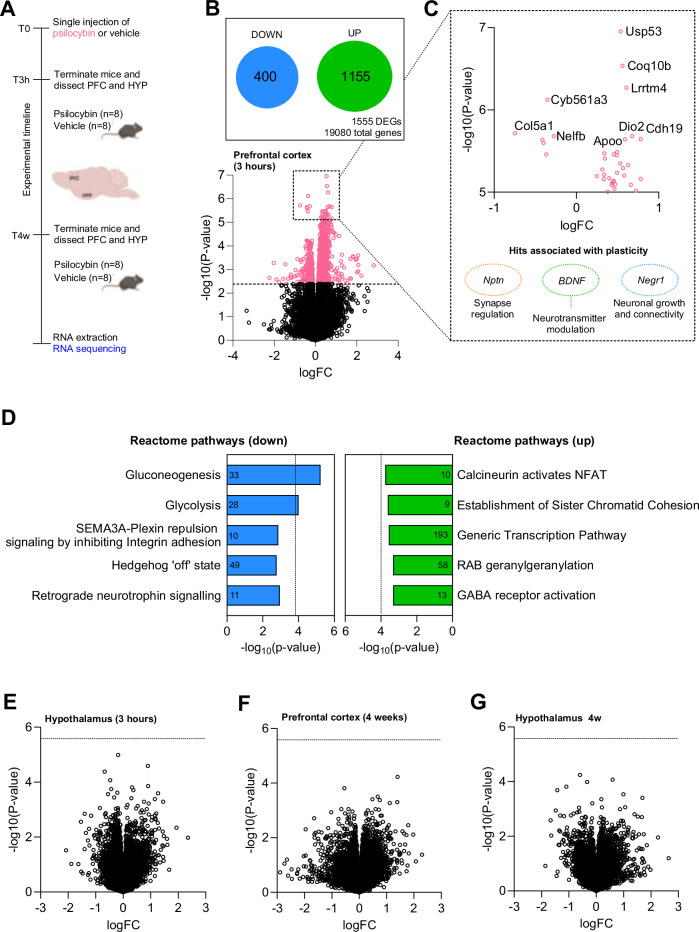
Acute and chronic hypothalamic and prefrontal cortex (PFC) transcriptomes following a single high dose of psilocybin. **A** Schematic overview of bulk RNA-seq study. Lean mice were split into two groups and received either a single dose of psilocybin (3 mg/kg) or a vehicle (*n* = 16 per group). Three hours following dosing, eight psilocybin-treated and eight vehicle-treated mice were sacrificed, and PFC and whole hypothalamus were dissected. The remaining mice were sacrificed 4 weeks after injection. **B** Schematic showing the effect of psilocybin in the PFC 3 h after administration. **C** Top 10 significantly differentially expressed genes in PFC (3 h after administration of psilocybin). Points in pink represent genes with p.adj < 0.05. **D** Reactome pathways for upregulated and downregulated genes in PFC 3 h after administration of psilocybin. **E** Volcano plot showing the effect of psilocybin in the hypothalamus 3 h after administration of psilocybin vs. vehicle. **F** Volcano plot showing the effect of psilocybin in PFC 4 weeks after administration of psilocybin vs. vehicle. **G** Volcano plot showing effect in the hypothalamus for psilocybin-treated versus vehicle-treated mice 4 weeks after administration. Cut off for volcano plots **B**, **E**, **F**, and **G** calculated as 0.05/19080 number of tested genes.

The widespread transcriptional effects of psilocybin on the brain have not been comprehensively studied. In rats, a single dose of psilocybin rapidly induced gene expression in the PFC compared to the hippocampus [35], with an increase in expression of genes associated with neuronal plasticity (*Psd95*) and neuron activation (*c-Fos*). To our knowledge, the transcriptional changes in the hypothalamus, following administration of psilocybin have not yet been characterized. To assess the transcriptional response to psilocybin (acute and long-term effect), chow-fed mice were randomized into two groups (psilocybin treated, or vehicle). Given that a single dose of psilocybin is reported to cause structural changes to dendritic spines in the cortex that persists even after a month [61], we dissected the hypothalamus and PFC after both 3 h and 4 weeks following a single administration of psilocybin (Fig. 4A). Significant differential expression was observed in the PFC 3 h following treatment, with 400 upregulated and 1155 downregulated genes (Fig. 4B). Among the differentially expressed genes were neuroplastin (*nptn*), brain-derived neurotrophic factor (*BDNF*) and neuronal growth regulator 1 (*Negr1*)—genes associated with cognition, neuronal growth, plasticity and obesity (Fig. 4C) [62–64]. Reactome pathways upregulated in the PFC include gluconeogenesis and glycolysis (Fig. 4D). Given the expression of 5-HT_2A_R in the hypothalamus, we were surprised to find no effect of psilocybin on the hypothalamic transcriptome at either 3 h of 4-week post-administration of psilocybin (Fig. 4E–G). [65].

## Discussion

Obesity is a relatively treatment-resistant condition; many patients do not reap the benefits of pharmacological or lifestyle-based interventions [66]. Following dietary, therapeutic, and even surgical interventions, homeostatic mechanisms defend the elevated body weight, hindering weight loss efforts [67]. Recent advancements in the development of anti-obesity medications have given rise to new therapies that safely deliver sizeable weight loss, but require a chronic treatment regime for weight loss maintenance [68, 69]. Thus, the lofty vision of therapeutically lowering body weight in a manner that persists remains unmet.

The psychedelic renaissance has sparked new hope for the treatment of many behavioral and neuropsychiatric conditions that have—similarly to the management of obesity—shown to be extremely treatment-resistant [7]. For the treatment of addiction [70] and depression [10], a combination of psilocybin and psychotherapy has shown to produce clinical remission that, in some cases, persists for years. Given the neurobiological and behavioral similarities between obesity and addiction, we conducted the first preclinical evaluation of psilocybin for obesity and binge-like eating behavior in mouse models of genetic and diet-induced obesity. We demonstrate that neither a single ‘high’ dose of psilocybin (3 mg/kg) nor a daily microdosing approach induces metabolic or behavioral changes in mouse models of obesity and binge-eating. During our studies, we did not observe any adverse effects of psilocybin, suggesting that the doses and treatment applied here are safe and well tolerated.

Psilocybin’s unique ability to enhance cognitive flexibility [52] has made it a relevant target for the treatment of depression, and disorders with compulsive symptomatology [57]. Deficits in cognitive flexibility are associated with alcohol use disorder [71], obesity [72], and eating disorders [73]—hence psilocybin could prove to be beneficial for the management of these conditions. In the present study, we found no evidence that psilocybin could provide a neural background that supports weight loss, maintenance of weight loss, or reductions in binge eating. We did observe a reduction in sucrose preference following administration of psilocybin, which supports that it could be useful for the treatment of compulsive behavior or for dampening excessive consumption of sugar and sweet foods in susceptible individuals.

The question remains—are rodent models applicable for translational evaluation of 5-HT_2A_R-targeting psychedelics as tools for weight management and eating disorders? A similar question was posed in a study assessing the effect of psilocybin on an animal model of alcohol use disorder—where no effect was found [56]. A later study by the same authors combined animal models with genetic manipulation of prefrontal cortex glutamate receptors and elucidated a deeper understanding of psilocybin’s effect [57]. Based on the findings of the present study, it may be premature to discard the use of psilocybin for the treatment of obesity and disordered eating in humans. Psilocybin may be particularly efficacious in subpopulations of obese patients with a disease etiology rooted in disordered eating. The first clinical trials assessing the use of psilocybin for the treatment of anorexia nervosa are starting, based on this very premise [74] (clinical trial numbers: NCT04661514, NCT04052568, and NCT04505189).

Given psilocybin’s virtue of being a 5-HT_2A_R agonist (via psilocin) and a weak agonist of 5-HT_2C_R, it was surprising that psilocybin had no impact on food intake or body weight in the wide variety of conditions tested, including lean mice, diet-induced-obese mice, *ob/ob* mice and melanocortin-receptor 4 knockout mice. Obesity-susceptible mice have increased 5-HT_2A/2C_ receptor density in the ventromedial hypothalamus (VMH) [75] and serotonin release from the VMH is reduced in animal models of obesity. Perhaps psilocybin’s effect on the homeostatic regulation of food intake is limited, as an expression of 5-HT_2A_R in the hypothalamus is relatively low [76]. We observed no short or long-term changes in transcriptional programs in the hypothalamus 3 h or 4 weeks after peripheral administration of psilocybin. In contrast, substantial transcriptional changes were observed in the prefrontal cortex, 3 h after administration of psilocybin. These acute changes included alterations in canonical gene programs related to cellular substrate metabolism, signaling, and neuroplasticity. The relationship between obesity and the dorsolateral prefrontal cortex has been established; lower activation is observed in obese patients compared to lean [77], but this has never been targeted therapeutically. Psilocybin presents a novel approach for altering executive function to reduce impulsive food intake behavior. However, the observed transcriptional changes did not endure, and as such, cannot explain the long-lasting benefits of a single administration of psilocybin.

In summary, our preclinical evaluation of psilocybin for the treatment of obesity and binge-eating disorders demonstrates that psilocybin does not lower body weight and food intake in obese mice, but a single high dose of psilocybin does lower the preference for sucrose in lean mice. This effect may be linked to acute neuroplastic changes in the prefrontal cortex and suggests that psilocybin could have therapeutic value for lowering the hedonic value of certain hyperpalatable foods. Despite the decreased sucrose preference in lean mice, neither a single high-dose, nor microdosing approach was sufficient for reducing body weight and food intake, nor was a single high-dose able to attenuate binge eating behavior. Given the uncertainties about psilocybin’s therapeutic benefit in this context, further studies are required. In order to fully evaluate psilocybin, or similar 5-HT_2A_R targeting psychedelics, for perturbed eating behavior and weight management, a combinatorial approach— comprising psychedelic therapy, psychotherapeutic support, and lifestyle intervention could be considered.

## Supplementary information

Supplemental Material

## Data Availability

Data generated from the bulk RNA-seq study are available in GEO under accession number GSE209859.

## Code availability

The source code used to analyze the bulk RNA-seq data is available at https://github.com/CBMR-Single-Cell-Omics-Platform/Acute-and-long-term-effects-of-psilocybin-on-energy-balance-and-feeding-behavior-in-mice.
