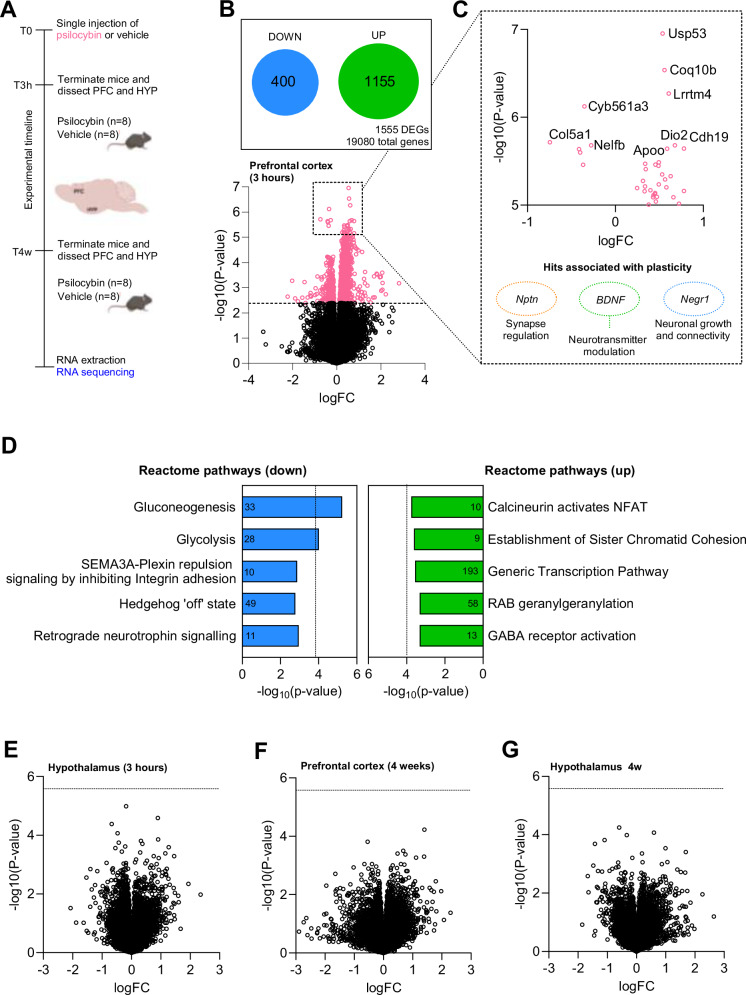# Correction: Acute and long-term effects of psilocybin on energy balance and feeding behavior in mice

**DOI:** 10.1038/s41398-025-03729-1

**Published:** 2025-11-06

**Authors:** Nicole Fadahunsi, Jens Lund, Alberte Wollesen Breum, Cecilie Vad Mathiesen, Isabella Beck Larsen, Gitte Moos Knudsen, Anders Bue Klein, Christoffer Clemmensen

**Affiliations:** 1 Novo Nordisk Foundation Center for Basic Metabolic Research, Faculty of Health and Medical Sciences, University of Copenhagen, Copenhagen, Denmark; 2 Neurobiology Research Unit, Copenhagen University Hospital, Rigshospitalet Copenhagen, Denmark; 3 Faculty of Health and Medical Sciences, University of Copenhagen, Copenhagen, Denmark

**Keywords:** Physiology, Molecular neuroscience, Diseases

Correction to: *Translational Psychiatry* 10.1038/s41398-022-02103-9, published online 11 August 2022

In the originally published version of this article, Figure 4 contained an error in the RNA-seq analysis. During data processing, the subtraction order for the treatment groups was inadvertently reversed (vehicle was subtracted from psilocybin-treated samples, instead of psilocybin-treated from vehicle). This error affected the plotted log fold-change values but does not alter the interpretation of the results, which were discussed only in general terms, nor does it impact the overall conclusions of the manuscript.

The corrected Figure 4 is shown below, and the associated online analysis code has been updated accordingly. The original article has been corrected.