# Supplementary material for: Acute and long-term effects of psilocybin on energy balance and feeding behavior in mice
Source: Transl Psychiatry. 2022 Aug 11;12:330. doi: 10.1038/s41398-022-02103-9 (PMC9372155; doi:10.1038/s41398-022-02103-9)
Supplement: Supplementary file 1 — Supplemental Material [file 41398_2022_2103_MOESM1_ESM.pdf]

Supplementary Information for

Acute and long-term effects of psilocybin on energy balance and feeding behavior in mice

Nicole Fadahunsi[1], Jens Lund[1], Alberte Wollesen Breum[1], Cecilie Vad Mathiesen[1], Isabella Beck Larsen[1], Gitte Moos Knudsen[2,3], Anders Bue Klein[1], Christoffer Clemmensen[1*]

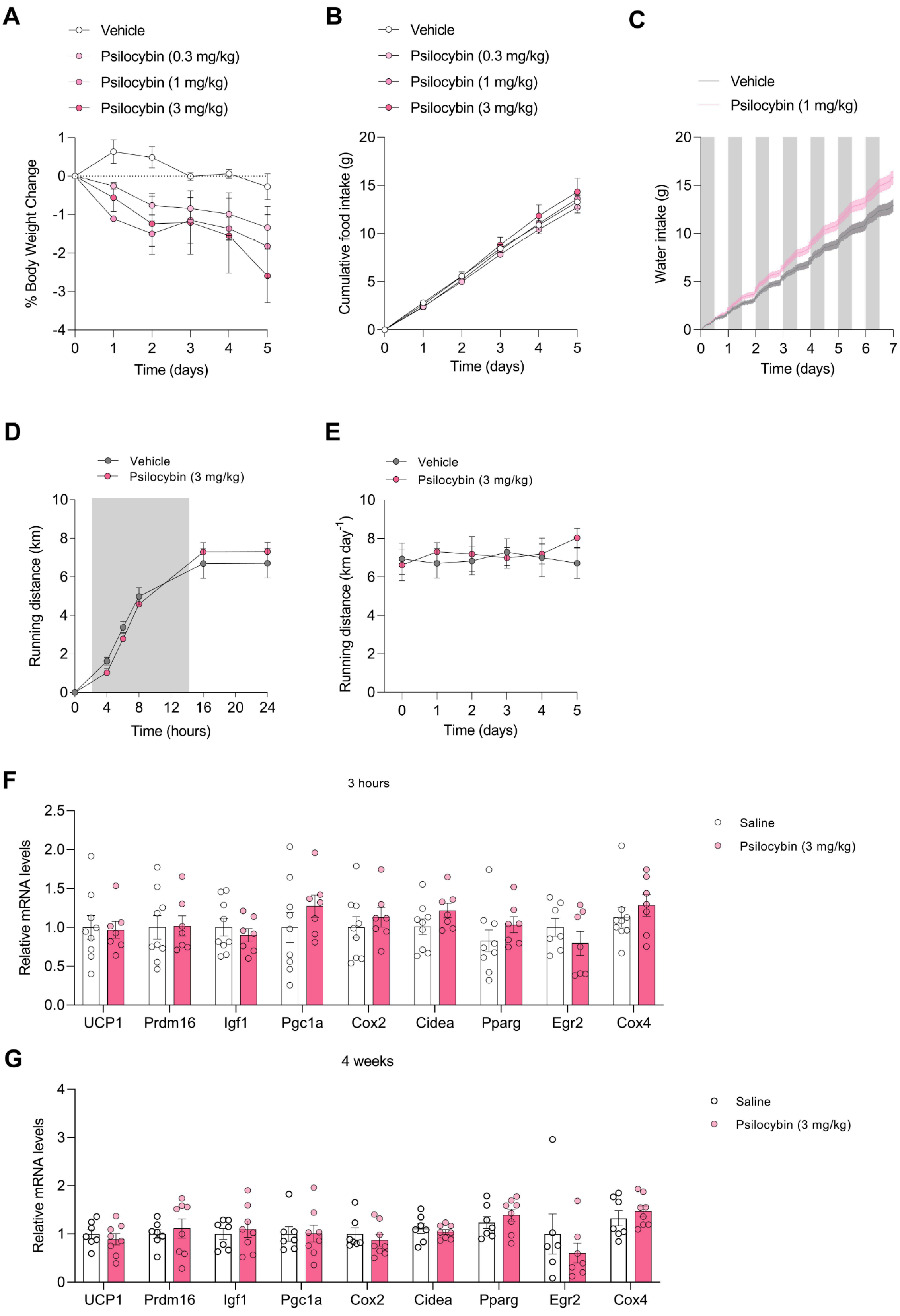
A
Vehicle
Psilocybin (0.3 mg/kg)
Psilocybin (1 mg/kg)
Psilocybin (3 mg/kg)
% Body Weight Change
Time (days)
B
Vehicle
Psilocybin (0.3 mg/kg)
Psilocybin (1 mg/kg)
Psilocybin (3 mg/kg)
Cumulative food intake (g)
Time (days)
C
Vehicle
Psilocybin (1 mg/kg)
Water intake (g)
Time (days)
D
Vehicle
Psilocybin (3 mg/kg)
Running distance (km)
Time (hours)
E
Vehicle
Psilocybin (3 mg/kg)
Running distance (km day-1)
Time (days)
F
3 hours
Saline
Psilocybin (3 mg/kg)
Relative mRNA levels
UCP1
Prdm16
Igf1
Pgc1a
Cox2
Cidea
Pparg
Egr2
Cox4
G
4 weeks
Saline
Psilocybin (3 mg/kg)
Relative mRNA levels
UCP1
Prdm16
Igf1
Pgc1a
Cox2
Cidea
Pparg
Egr2
Cox4

Fig. S1. Effect of single subcutaneous administration of psilocybin in diet-induced obese mice. Diet-induced obese mice received a single subcutaneous injection of psilocybin at 0.3 mg/kg, 1 mg/kg, 3 mg/kg or vehicle (n=6). A) Body weight and food intake were recorded in the five days after administration, no significant difference in body weight between any groups after five days of administration B) Food intake. C) Water intake following a single injection of 1 mg/kg psilocybin or vehicle (n=7 per group) to a separate cohort of mice in indirect calorimetry cages (mice on HFD). D) Effect of a single high dose of psilocybin (3 mg/kg) or vehicle on voluntary wheel behavior (24 hours), (n=8 per group). I) Running distance in the five days following injection with psilocybin (3 mg/kg) or vehicle (n=8 per group). E) F) Relative gene expression of thermogenic genes in BAT, 3 hours after injection of psilocybin (3 mg/kg) or vehicle (n=8 per group). G) Relative gene expression of thermogenic genes in BAT, 4 weeks after injection of psilocybin (3 mg/kg) or vehicle (n=8 per group). Data are presented as mean ± SEM.

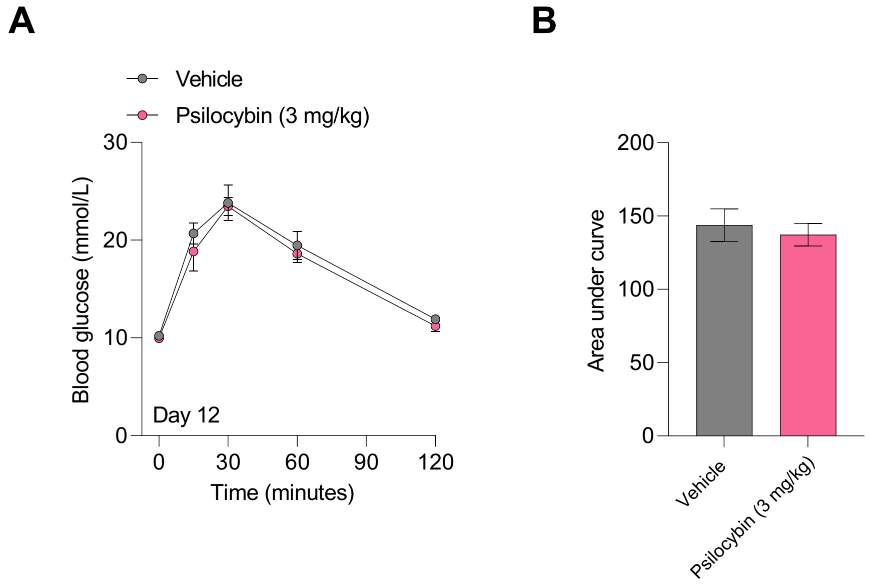

Fig S2. Effect of a single administration of psilocybin on glucose homeostasis in diet-induce obese mice. On the final day (day 12) of the 'food intake and body weight' study in DIO mice, a glucose tolerance test was performed. Mice were fasted for five hours before receiving a single intraperitoneal injection of 1.5 g/kg glucose dissolved in isotonic saline. A handheld glucometer (Contour XT, Bayer) was used to measure blood glucose in blood from the tail tip both before (0 minutes) and 15, 30, 60 and 120 minutes after injection (n=8 per group). A) Glucose tolerance test B) AUC of glucose tolerance test. Data analyzed by two-way ANOVA (A) and unpaired t-test (B).Data are presented as mean ± SEM.

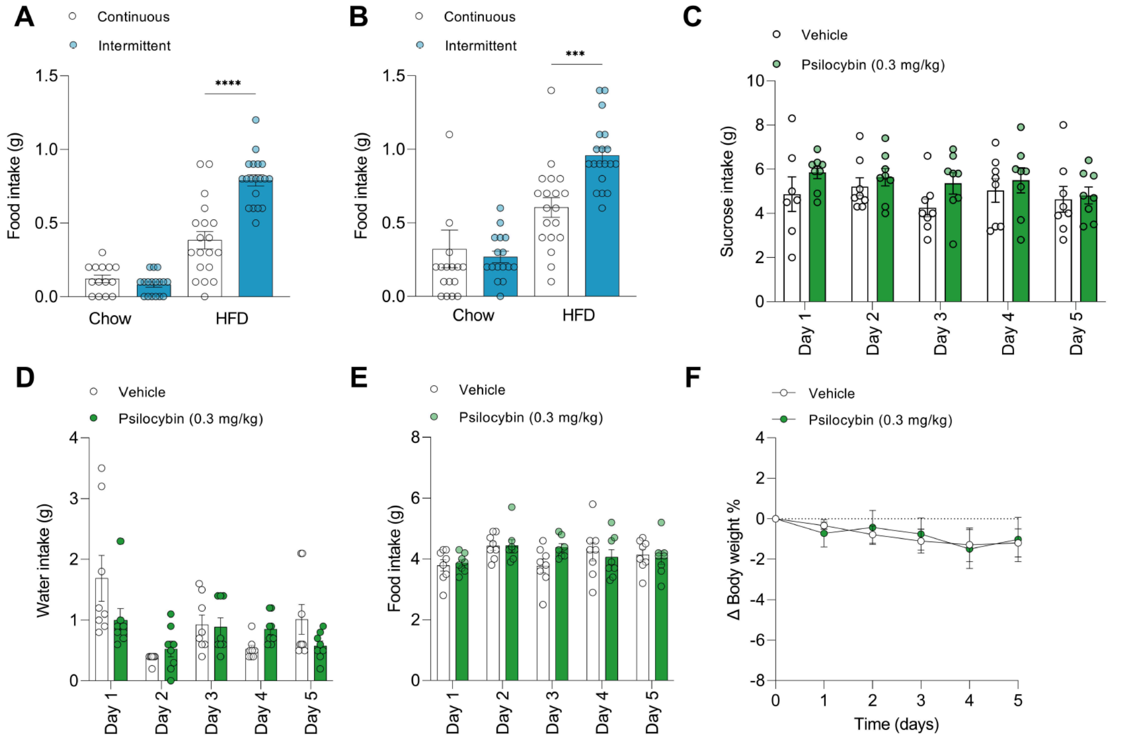

Fig. S3. Binge-eating paradigm and sucrose preference test with microdoses of psilocybin. A) Binge cycle 2. B) Binge cycle 3 (n=20 mice in continuous group, n=19 mice in intermittent group). 9 data points excluded from binge cycle 2, and 6 from binge cycle 3 due to scale error. C) Sucrose intake following daily injection of psilocybin (0.3 mg/kg) or vehicle (n=8 per group). D)**P*<0.05, ***P*<0.01, ****P*<0.001, *****P*<0.0001. (A,B, C, D, E) analyzed by unpaired t-test F) Analyzed with two-way ANOVA. Data are presented as mean ± SEM,
